# Systemic response to rupture of intracranial aneurysms involves expression of specific gene isoforms

**DOI:** 10.1186/s12967-019-1891-6

**Published:** 2019-05-02

**Authors:** Michal Korostynski, Marcin Piechota, Rafal Morga, Dzesika Hoinkis, Slawomir Golda, Magdalena Zygmunt, Tomasz Dziedzic, Marek Moskala, Agnieszka Slowik, Joanna Pera

**Affiliations:** 1 Department of Molecular Neuropharmacology, Institute of Pharmacology, Polish Academy of Sciences, ul. Smetna 12, 31-343 Kraków, Poland; 2 Department of Neurosurgery and Neurotraumatology, Faculty of Medicine, Jagiellonian University Medical College, ul. Botaniczna 3, 31-503 Kraków, Poland; 3 Intelliseq sp. z o.o., ul. Chabrowa 12/3, 31-335 Kraków, Poland; 4 Department of Neurology, Faculty of Medicine, Jagiellonian University Medical College, ul. Botaniczna 3, 31-503 Kraków, Poland

**Keywords:** Subarachnoid hemorrhage, Intracranial aneurysm, RNAseq, Monocytes, Lymphocytes

## Abstract

**Background:**

Rupture of an intracranial aneurysm (IA) causes a systemic response that involves an immune/inflammatory reaction. Our previous study revealed a downregulation of genes related to T lymphocytes and an upregulation of genes related to monocytes and neutrophils after IA rupture. It remains unknown whether that resulted from alterations in transcription or cell count. We sought to characterize the systemic response to IA rupture through analysis of transcript expression profiles in peripheral blood cells. We also investigated effects of IA rupture on the composition of mononuclear cells in peripheral blood.

**Methods:**

We included 19 patients in the acute phase of IA rupture (RAA, first 72 h), 20 patients in the chronic phase (RAC, 3–15 months), and 20 controls. Using deep transcriptome sequencing, we analyzed the expression of protein-coding and noncoding RNAs. Expression levels, transcript biotypes, alternative splicing and other features of the regulated transcripts were studied. A functional analysis was performed to determine overrepresented ontological groups among gene expression profiles. Flow cytometry was used to analyze alterations in the level of mononuclear leukocyte subpopulations.

**Results:**

Comparing RAA and controls, we identified 491 differentially expressed transcripts (303 were downregulated, and 188 were upregulated in RAA). The results indicate that the molecular changes in response to IA rupture occur at the level of individual transcripts. Functional analysis revealed that the most impacted biological processes are related to regulation of lymphocyte activation and toll-like receptor signaling pathway. Differences between RAC and controls were less prominent. Analysis of leukocyte subsets revealed a significantly decreased number of CD4+ lymphocytes and increase of classical and intermediate monocytes in RAA patients compared to controls.

**Conclusions:**

IA rupture in the acute phase strongly influences the transcription profiles of peripheral blood cells as well as the composition of mononuclear cells. A specific pattern of gene expression alteration was found, suggesting a depression of lymphocyte response and enhancement of monocyte activity.

**Electronic supplementary material:**

The online version of this article (10.1186/s12967-019-1891-6) contains supplementary material, which is available to authorized users.

## Background

Rupture of an intracranial aneurysm (IA) causes subarachnoid hemorrhage (SAH), which is burdened with high mortality and high disability rates [1, 2]. This poor prognosis is driven by direct neurological sequels of IA rupture and systemic complications resulting from bed-ridden patient status and infections. The latter are associated with immunodepression related to central nervous system acute injury [3, 4]. Molecular mechanisms underlying systemic responses to SAH remain poorly characterized. A range of changes in levels of different molecules, blood cells counts and their function in peripheral blood was reported [5–7].

Using a microarray approach, we previously showed that IA rupture influences the transcriptional profiles of peripheral blood cells, and the pattern of these changes suggests a depression in lymphocyte response with enhancements in monocyte and neutrophil activities [8]. In patients with poor outcomes, the differences in levels of cell-related transcripts were significantly more prominent. However, whether observed changes in genes transcriptions reflected changes in blood cell counts, cell activation levels or both remained open. There is an increasing body of evidence that SAH is associated with a dysregulation of lymphocyte subsets [9–11] and—particularly in patients with SAH-related complications—there are significant changes in lymphocyte and monocyte counts and functions [5]. Studies on ischemic stroke also showed its strong influence on the number and activity of monocyte and lymphocyte subclasses and their associations with clinical course [12, 13].

In the current study, we sought to investigate the systemic effects of aneurysmal SAH through analysis of gene expression profiles in peripheral blood cells using deep transcriptome sequencing. We performed a global analysis of protein coding and noncoding gene variants regulated by SAH. We also investigated the expression of specific gene variants that may lead to production of functionally distinct protein isoforms and influence systemic response to IA rupture. Moreover, we analyzed subpopulations of mononuclear leukocytes in peripheral blood after IA rupture. These SAH-related changes were evaluated both in the acute and in the chronic phase and were compared with the changes in control subjects.

## Methods

### Patients

Consecutive patients with aneurysmal SAH were prospectively recruited in University Hospital, Krakow in 2014 and 2015. Patients with head trauma, vasculitis, arteriovenous malformations, hematological disorders, and known malignancies were excluded. Two independent patients groups were analyzed: acute (first 72 h after IA rupture) and chronic (3–15 months after SAH). Control subjects (C) were recruited from patients who suffered from headaches. All subjects were Caucasian. Informed consent was obtained from all participants (or their guardians). The local ethics committee approved the study. Differences between groups were analyzed using χ^2^ and Fisher exact tests or Mann–Whitney *U* test where appropriate, p < 0.05 was considered statistically significant.

### Blood collection and RNA extraction

Venous whole blood was collected before neurosurgical or endovascular interventions in PAXgene Blood RNA Tubes (PreAnalytiX, GmbH, Switzerland). Total RNA was isolated using PAXgene Blood RNA Kit (PreAnalytiX) following the manufacturer’s protocol. RNA concentrations were measured using a NanoDrop ND-1000 Spectrophotometer (NanoDrop Technologies, Montchanin, DE), and RNA quality was determined by chip-based capillary electrophoresis utilizing an RNA 6000 Nano LabChip Kit and an Agilent Bioanalyzer 2100 (Agilent, Palo Alto, CA).

### Whole-transcriptome sequencing

Before library generation with TrueSeq Stranded Total RNA Library Prep (Illumina, San Diego, CA), ribosomal RNA was removed with Globin-Zero Gold Removal Kit (Illumina). Whole transcriptome libraries were sequenced on a HiSeq 4000™ (Illumina) with the following parameters: PE 150 (paired ends) and 45 M clean reads which gave a minimum of 13.5 Gb of raw data per sample. The RNA-seq data were submitted to the NCBI Sequence Read Archive (SRA): SRP150595.

### RNA-seq data analysis and transcript classification

The quality of NGS data was verified using FastQC. The RNA-seq reads were aligned to GRCh37.p13 using Hisat2 2.0.5. The transcript FPKM (Fragments Per Kilobase of transcript per Million fragments mapped) levels were quantified using the Cufflinks v2.2.1 and GTF from the Ensembl gene database. Statistical significance was analyzed using one-way analysis of variance (ANOVA) on log_2_(1 + x) values which have normal continuous distribution [14]. The false discovery rate (FDR) was estimated using the Benjamini–Hochberg method. All statistical analyses were performed using R software v3.4.3. Transcript annotation and classification was performed using the BioMart interface for the Ensembl gene database.

### Functional classification of differentially expressed genes

The gene annotation tool Enrichr was used to identify overrepresented ontological groups among the gene expression patterns and to group the genes into functional classifications [15]. Overrepresented terms (Wiki Pathways 2016) were defined as having at least three transcripts and p < 0.05 per Fisher’s exact test. For cell-type enrichment of mRNA, the Enrichr cell types (Human Gene Atlas) module was used. The identification of overrepresented transcription factor binding sites (TFBSs) in the regulatory regions of genes was performed using the ChIP-seq data (ChIP Enrichment Analysis, ChEA2016) module implemented in the Enrichr online resource. Gene symbol lists were submitted, and default parameters were used.

### Validation of NGS results using quantitative PCR (qPCR)

Reverse transcription was performed using Omniscript Reverse Transcriptase (Qiagen Inc.). The qPCR reactions were performed using isoform specific TaqMan^®^ probes or designed by the Custom TaqMan^®^ Assay Design Tool (Life Technologies) and were run on the CFX96 Real-Time system (BioRad). Overall, 17 qPCR assays for 8 genes (*HEATR1*, *ACBD6*, *CCND2*, *PLEKHA1*, *ELF2*, *CFLAR*, *TRAF1* and *PPP1R16B*) have been used. The list of assays including target transcripts is presented in Additional file 1: Table S1. Expression of the hypoxanthine–guanine phosphoribosyltransferase 1 (*HPRT1*) transcript was quantified to control for variations in cDNA concentrations. The abundance of each RNA was calculated as 2^−(threshold cycle)^. Data were analyzed using one-way ANOVA followed by Tukey’s multiple comparisons test.

### L/MN index

A lymphocyte-to-monocyte-and-neutrophil index (L/MN index) was measured as previously described [8]. Based on RNAseq results, the L/MN index was calculated as the ratio of the mean folds of standardized expression levels of lymphocyte-related genes (*BCL11B*, *CCR7*, *CD2*, *CD27*, *CD3D*, *CD3E*, *CD8A*, and *KLRB1*) to those of monocyte-and-neutrophil-related genes (*ANXA3*, *ARG1*, *CD14*, *GYG1*, *FCGR1A*, *FCGR2A*, *IRAK3*, and *MMP9*). The abundance levels of the specific IA-altered transcriptional variants of the selected genes were used in the analysis (Additional file 2: Table S2). T-tests with Welch’s correction were performed on L/MN values between the groups of patients.

### Analysis of subsets of mononuclear leukocytes by flow cytometry

Blood was collected in EDTA-tubes in the morning after admission. White blood cell (WBC) count and differential were determined by the clinical laboratory. Monocyte and lymphocyte subpopulations were analyzed in whole blood by flow cytometry using BD FACSCanto II with Diva software v 6.1.2 (BD Biosciences San Jose, CA). Blood samples were incubated with a premixed antibody cocktail for 15 min, then erythrocytes were lysed with BD FACS Lysing Solution (BD Biosciences). The following fluorochrome-conjugated monoclonal antibodies were used (all antibodies were mouse IgG1): FITC-CD14 (MφP9; BD Biosciences), PE-CD16 (3G8; Beckman Coulter, Miami, FL), PC5-CD3 (UCHT1; Beckman Coulter), FITC-CD4 (SK3; BD Biosciences), PE-CD8 (SK1; BD Biosciences), PC5-CD33 (D3HL60,251; Beckman Coulter), and invariant natural killer T (*i*NKT) Cell (6B11; BD Pharmingen, San Jose, CA). Antibody-capture beads (CompBeads, BD Biosciences) were used for single-color compensation controls for each reagent used in the study. Lymphocyte and monocyte subsets were identified based on forward scatter (FSC) vs side scatter (SSC) and positive expression of their respective markers. Absolute numbers of lymphocyte and monocyte subsets were calculated by multiplying the total WBC with the total percentage of each cell population. First, we excluded doublets using FSC area vs FSC height and analyzed only singlets. Lymphocyte populations were analyzed by gating using FSC and SSC. The lymphocyte T-population was analyzed by gating CD3+ cells and on the next dot plot—SSC vs CD3. From this gate, T lymphocytes were selected and further divided into: CD4+CD8−, CD4−CD8+, CD4+CD8+, and CD4−CD8− lymphocytes. In addition, using an anti-*i*NKT cell antibody, we identified CD3+ *i*NKT cells. Monocyte populations were analyzed by gating using FSC and SSC. Next, from the CD33+ and CD14+ cell gate and the gate on the SSC vs CD14 dot plot, we extracted the monocyte population. On the next dot plot (CD14 vs CD16), three monocyte subsets were distinguished: CD14++CD16− (classical), CD14++CD16+ (intermediate), and CD14+CD16++ (nonclassical).

## Results

### Profile of transcriptomic alterations induced by IA rupture

The study comprised 19 patients in the acute phase of SAH (RAA), 20 patients in the chronic phase (RAC), and 20 control subjects. Their clinical characteristics are summarized in Table 1. In 8 patients ruptured IA was closed by clipping, in remaining 11—by coiling. During hospitalizations, among RAA patients we noticed: one patient developed symptomatic vasospasm, 1 rebled and required external drainage, 1 had urinary tract infection, 1—bronchitis with subsequent pneumonia, 1—pneumonia accompanied by heart failure, and 1 who developed sepsis and multiorgan failure. Two latter patients died. Transcriptional patterns found in these patients did not differ significantly from the rest of the RAA group, and their exclusion (as a subgroup or individually) from the analyses did not influenced significantly obtained results. Also the same was true for the cell subsets analyses.

**Table 1:** Clinical characteristics of the patients

	RAA (n = 19)	RAC (n = 20)	C (n = 20)	p RAA vs RAC	p RAA vs C	p RAC vs C
Median age (IQR), years	54 (48–62)	50 (41–56)	55 (50–60)	n.s.	n.s.	n.s.
Female, %	73.7	90.0	55.5	n.s.	n.s.	0.03
Hypertension, %	57.9	55.0	50.0	n.s.	n.s.	n.s.
Smoking, %	31.6	40.0	30.0	n.s.	n.s.	n.s.
Excessive drinking, %	0	5.0	10.0	n.s.	n.s.	n.s.
Aneurysms location						
Anterior circulation	17	16	–			
Posterior circulation	2	4	–			
Aneurysms number (number of patients)						
1	13	9	–			
2	6	9	–			
3	0	2	–			
Admission Hunt-Hess score (IQR)	2 (1–3)	–	–			
Admission GCS score (IQR)	15 (13–15)	–	–			
Admission WFNS score (IQR)	1 (1–3)	–	–			
Admission modified Fisher Scale (IQR)	3 (1–3)	–	–			

*RAA* acute phase of intracranial aneurysm (IA) rupture, *RAC* chronic phase of IA rupture, *C* control subjects, *IQR* interquartile range, *GCS* Glasgow Coma Scale, *WFNS* World Federation of Neurological Surgeons

We used NGS to examine gene expression at the level of specific transcriptional units. We measured FPKM for all the transcripts annotated in the GRCm38.p13 genome release using the Cufflinks package. A total of 94,239 transcripts corresponding to 35,529 annotated genes were detected at the threshold of FPKM ≥ 0.1.

The regulated transcripts were identified by one-way ANOVA with clinical status (RAA, RAC, C) as the factor. There were 491 differentially expressed transcripts (FDR < 0.001%). Comparing RAA and C groups, we found 403 differentially expressed transcripts (177 up- and 226 downregulated in RAA patients, fold change > 2). No significant differences were observed between RAC and C groups, and differences between RAA and RAC patients were noted for 268 transcripts (178 up- and 290 downregulated in RAA, fold change > 2). As blood samples from the RAC patients were obtained at different time points after IA rupture, we investigated the potential effects of the time lapse on gene expression profiles. We found two significant relationships (Spearman’s rank correlation coefficient R > 0.7, p < 0.01) between the time after SAH onset and the transcript expression levels. Both *SLC26A8* (ENST00000486155) and *BASP1*-*AS1* (ENST00000399760) were upregulated after IA rupture, and the level of transcript abundance decreased over time towards control levels (Additional file 3: Figure S1). The full list of regulated transcripts is presented in Additional file 4: Table S3.

### Functional annotations of the regulated transcripts

**Fig. 1:**
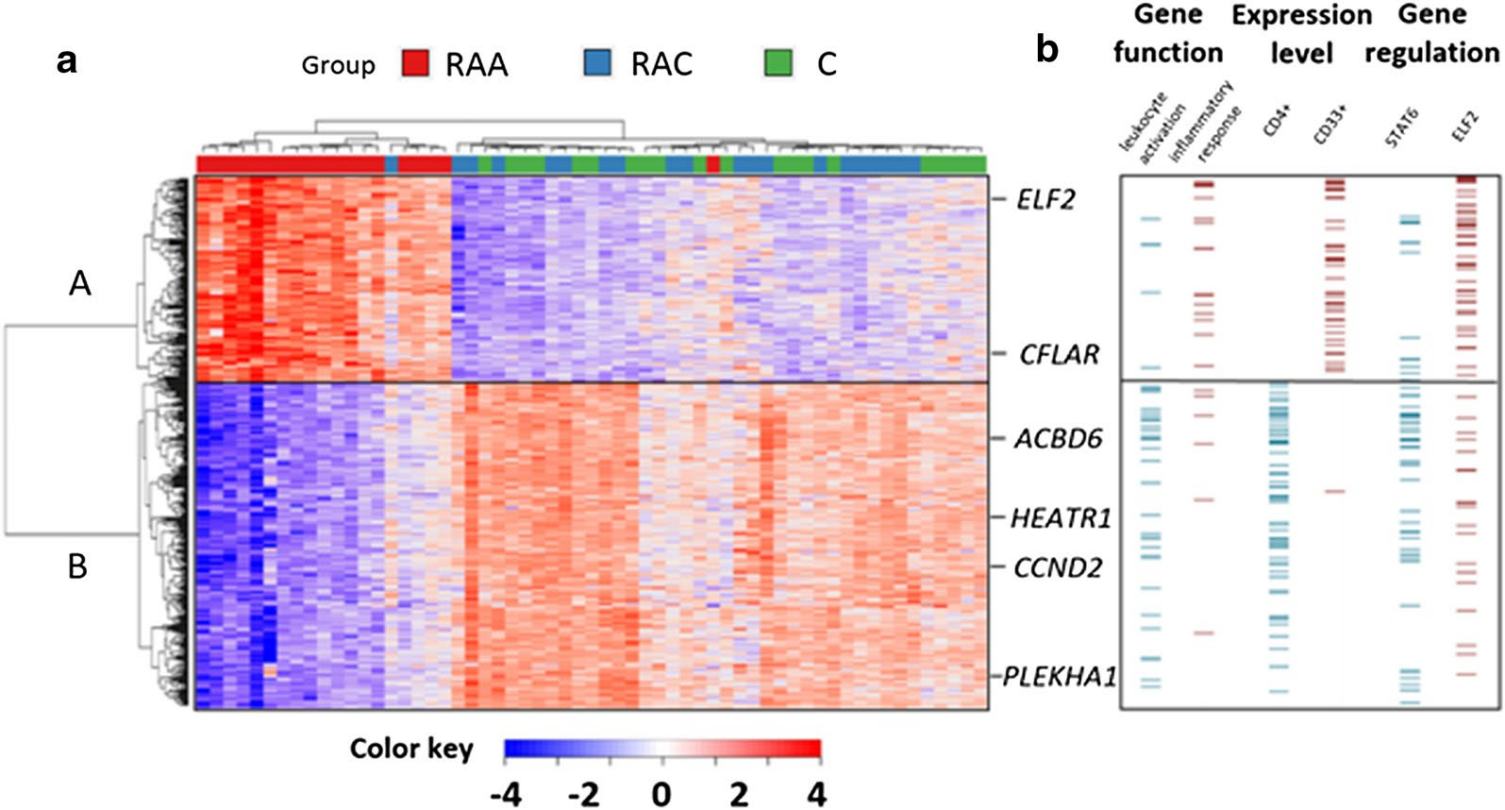
Profiling of transcriptional alterations in the peripheral blood cells of patients after IA rupture. **a** Hierarchical clustering of transcripts regulated in response to IA rupture. RNA-seq results are shown as a heat map and include 491 transcripts with a genome-wide significance (FDR < 0.01%) from one-way ANOVA for the factor of clinical status. Gene transcripts with altered RNA abundance levels are listed in Additional file 4: Table S3. Colored rectangles represent transcript abundance measured in the samples described above (RAA: acute phase of IA rupture, RAC: chronic phase of IA rupture, C: healthy controls). The intensity of the color is proportional to the standardized values (between −4 and 4) from each RNA-seq measurement, as indicated on the bar below the heat map image. Hierarchical clustering was performed with the R software using distance metric—correlation and the average linkage method. Two major gene transcription patterns in peripheral blood associated with IA rupture were arbitrarily denoted as A and B. Changes in the expression of six example genes (labeled on the right) were analyzed by using qPCR. **b** The analysis of gene expression patterns using data-mining methods. Functional enrichment among genes with similar expression profiles was identified by using the Enrichr analysis tool. Overrepresentation of functional links, TFBSs and expression in the particular cell types were indicated on the right. The examples of functional enrichment were indicated, and the complete results of the analysis are presented in Additional file 5: Table S4

Hierarchical clustering revealed two major gene transcription patterns in peripheral blood associated with IA rupture (arbitrarily described as A–B; Fig. 1a). These two groups revealed opposite patterns of up- and downregulated gene expression levels. Transcripts from the first cluster (303) showed decreased abundance in response to SAH. The second cluster includes genes (186) upregulated after IA rupture. Genes from each pattern were analyzed by the GO BP terms and Wiki Pathways 2016 categories. Functional analysis of the downregulated genes showed that the most overrepresented biological processes are those related to positive leukocyte activation (GO:0002694) and T cell differentiation (GO:0042110). Among Wiki pathways, those related to T-cell receptor signaling, inflammatory response and ribosomal proteins were enriched in the regulated genes. Examples of transcripts clustered to this group included *CD28*, *ITK* and *ZAP70*. Among the genes with increased mRNA abundance levels, transcripts connected with Toll-like receptor signaling pathway (GO:0002224) and activation of inflammatory response (GO:0006954) were overrepresented. Analysis of this pattern also revealed the enrichment of genes involved in IL1 and IL4 signaling pathways, including *IL1R1*, *IL1R* and *IRAK3*.

To investigate the mechanisms of transcriptional alterations in response to IA rupture, we searched for overrepresented transcription factor binding sites (TFBSs) in the regulatory regions of coexpressed transcripts. We exploited available ChIP-seq data (ChIP Enrichment Analysis, ChEA) using the module implemented in the Enrichr online resource. Promoters of downregulated transcripts exhibited a significant overrepresentation in the ChIP-seq signal for several transcriptional factors, including *STAT6* (adjusted p = 7 × 10^−8^, ChEA: “STAT6_20620947_ChIP-Seq_CD4_POS_T_Human”) and *RUNX* (adjusted p = 7.3 × 10^−7^, ChEA: “RUNX_20019798_ChIP-Seq_JUKART_Human”). Genes with increased mRNA abundance levels had a different set of putative transcriptional regulator binding sites, including *ELF2* (adjusted p = 0.01, ChEA: “Nerf2_26677805_Chip-Seq_MACROPHAGESS_Mouse”) and *SMRT* (adjusted p = 0.01, ChEA: “SMRT_22465074_ChIP-Seq_MACROPHAGES_Mouse”) (Fig. 1b).

Identification of cell types expressing genes in patterns A and B was carried out in reference to the Human Gene Atlas. A highly significant enrichment of transcripts from the pattern A are found in CD4+ (adjusted p = 1.9 × 10^−13^) and CD8+  T-cells (adjusted p = 3.8 × 10^−12^). Enriched transcripts from pattern B are found in the whole-blood (adjusted p = 1.2 × 10^−24^) and CD33+ myeloid cells (adjusted p = 2.2 × 10^−5^) (Fig. 1b). Details about functional classification, overrepresented TFBSs and cell-type enrichment are provided in Additional file 5: Table S4.

### Identification and classification of the altered transcriptional variants

**Fig. 2:**
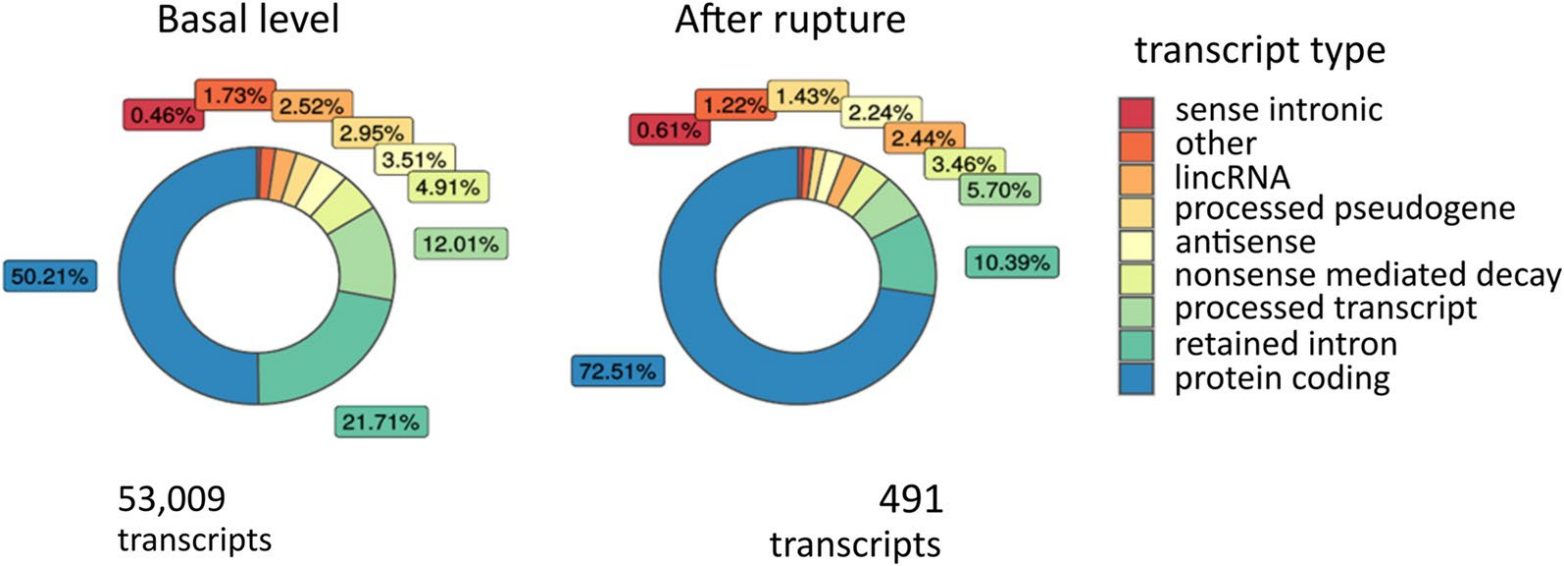
Distribution of biotypes among the transcripts altered in response to IA rupture. The charts presenting the proportion of transcripts biotypes at basal level (all the detected transcripts, FPKM < 0.5) and in response to IA rupture (regulated transcripts). The transcripts identified using RNAseq were classified using 29 known biotypes (based on Vega annotation; Additional file 4: Table S3)

To determine the diversity in the blood transcriptome, we classified the identified coding and noncoding transcripts by using 29 known biotypes (based on Vega annotation; Additional file 4: Table S3). 72.5% of the regulated transcripts were protein coding, compared with 50.2% of the transcripts expressed (FPKM > 0.5) at basal conditions (Fig. 2). The majority of noncoding, regulated transcripts were classified as retained intron (10.4%) and processed transcripts (5.7%) (vs 21.7% and 12% at basal conditions, respectively). Therefore, we further studied pre-mRNA levels of the genes altered in the RAA group. We compared the number of reads aligned to either exons or introns of the up- and downregulated genes. We found a pronounced decrease in the exon/intron ratio score for the downregulated genes.

The classifications of transcription alterations indicated different types of alternative expression of the differentially expressed genes. The analyses at the levels of gene isoforms, alternative exon splicing and alternative promoter usage were done by using Cuffdiff. We found 148 specific gene isoforms (annotated to 120 genes) altered in response to IA rupture (corrected p < 0.01, fold > 4). The majority of the transcripts differed between RAA and C (132) as well as RAA and RAC (125). Only 2 transcriptional isoforms (of *DEFA3* and *IFI27*) showed different levels between RAC and C groups. We did not find any statistically significant differences in alternative splicing or alternative promotors after the correction for number of tests performed (Additional file 6: Table S5).

### Validation of the alternative expression of genes

**Fig. 3:**
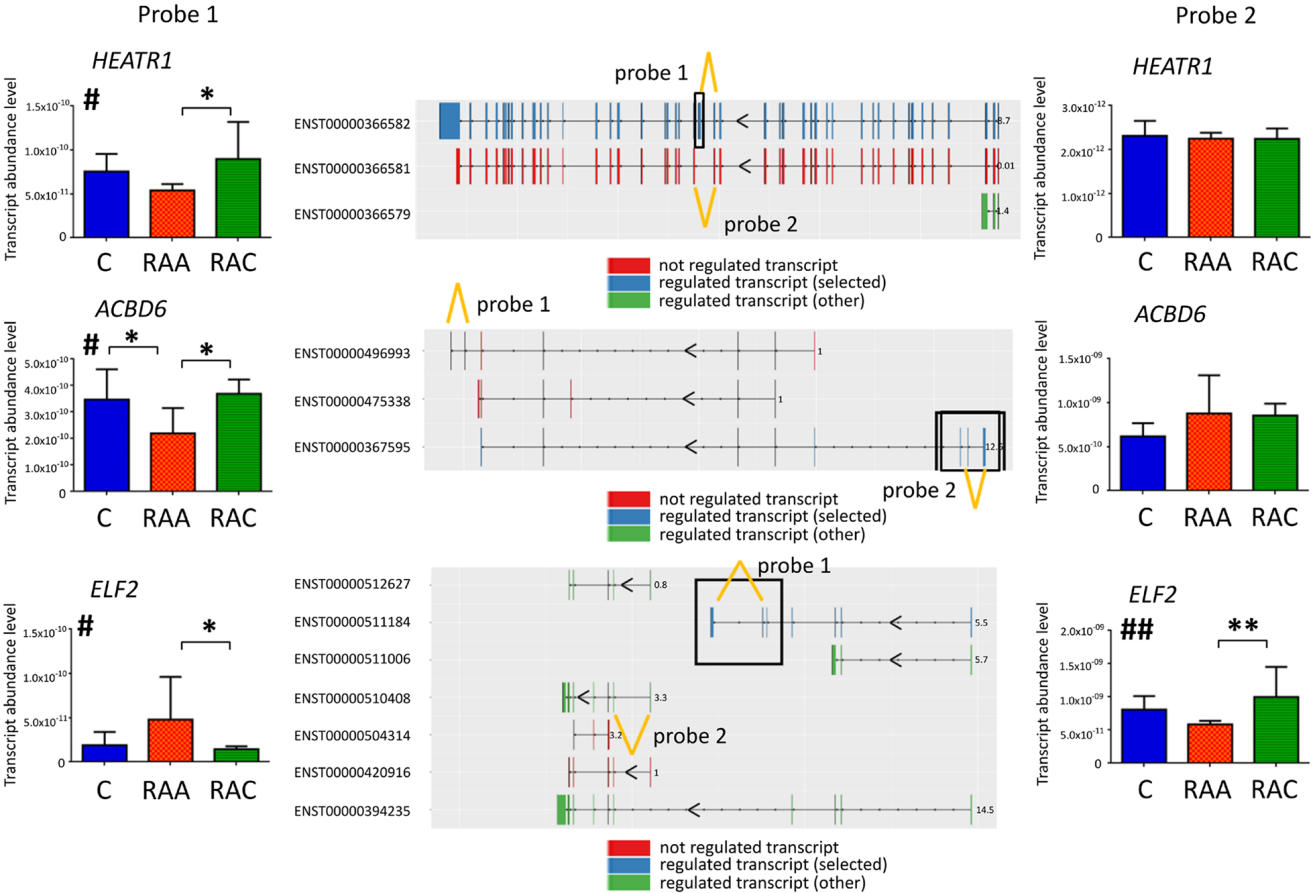
Validation of gene isoform-specific transcriptional alterations induced in response to IA rupture in peripheral blood. The expressions of *HEATR1*, *ACBD6* and *ELF2* transcripts were measured (Ensembl transcripts IDs are presented in the middle). qPCR analyses were performed to confirm isoform specific regulation using the same samples (n = 11–14). TaqMan probes allow for distinguishing between specific transcriptional variants. The assay locations are indicated by yellow lines. Bars indicate S.E.M, ^#^*p *< 0.05 from one-way ANOVA (factor of clinical status) followed by Tukey’s multiple comparisons test to identify differences between the groups (*p < 0.05, **p < 0.01)

We used qPCR to validate the results obtained by NGS. From 491 transcripts regulated after IA rupture, we selected 8 potential examples of isoform-specific gene expression (identified by Ensembl Transcript ID). The selected genes (Additional file 1: Table S1) were characterized by different profiles of regulation of the particular isoforms, each of the analyzed transcripts contains unique exons and a mean mRNA abundance level > 4 FPKM. The changes were measured using custom TaqMan assays designed to detect specific transcriptional variants. We found significantly different levels of expression for isoforms of *HEATR1* (ENST00000366582), *ACBD6* (ENST00000367595), *CCND2* (ENST00000261254), *PLEKHA1* (ENST00000368990), *ELF2* (ENST00000511184 and ENST00000510408), and *CFLAR* (ENST00000342795, ENST00000425030 and ENST00000309955). Opposite profiles of regulation between different isoforms of the same gene were revealed for *HEATR1*, *ACBD6* and *ELF2* (Fig. 3). In the cases of *PLEKHA1* and *CFLAR*, all analyzed variants showed similar regulation in response to IA rupture. For *CCND2,* only one TaqMan assay worked, and the results confirmed the downregulation detected by NGS. For *TRAF1* (ENST00000373887) and *PPP1R16B* (ENST00000299824) alteration profiles were similar to those obtained by RNA-seq but not statistically significant. Alterations in the expression of specific gene isoforms in response to IA rupture were reproducible.

### Effects of IAs rupture on subsets of peripheral mononuclear cells

**Fig. 4:**
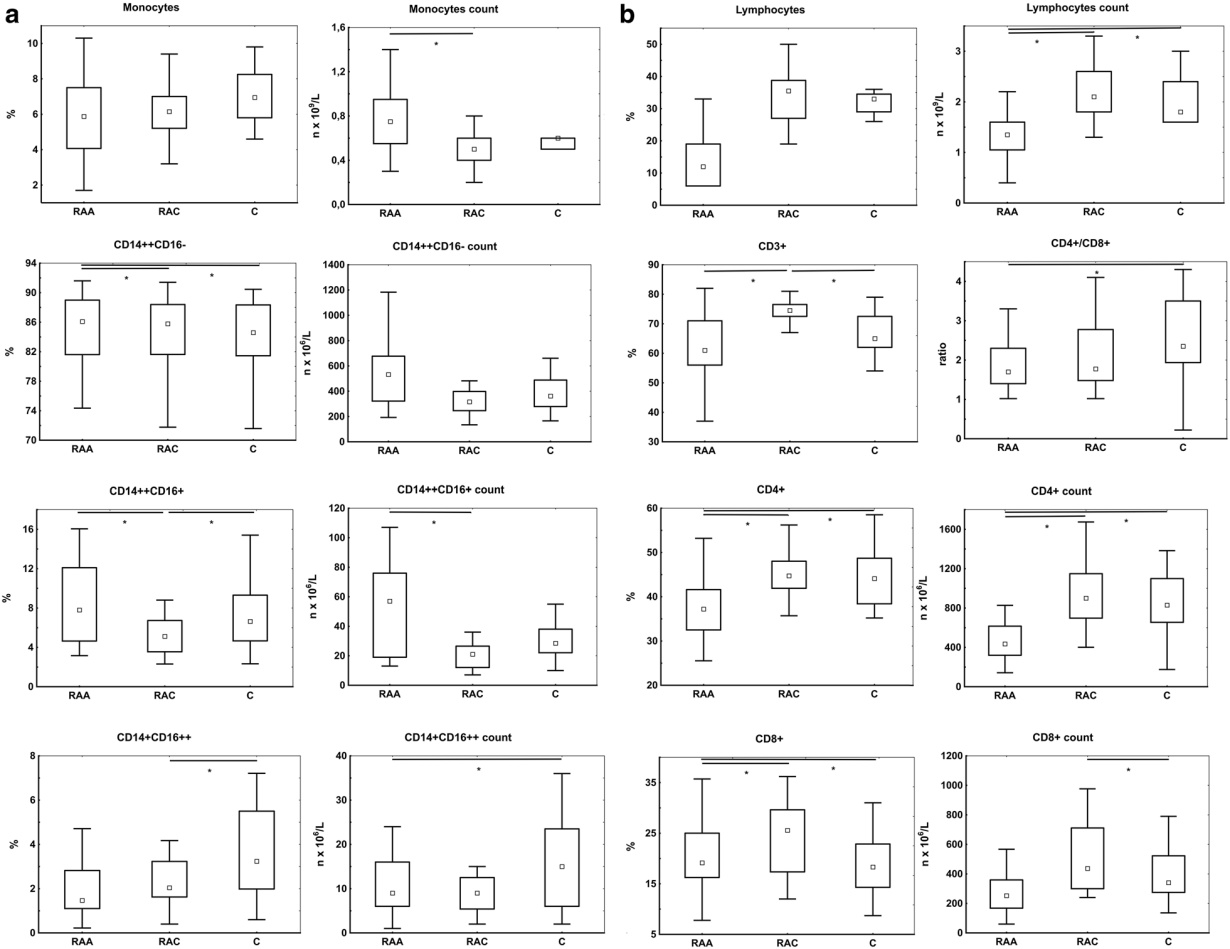
Effects of IA rupture on subsets of peripheral mononuclear cells. **a** Changes in count and proportions of monocytes and their subsets; **b** changes in count and proportions of lymphocytes and their subsets. Data are presented as medians and interquartile ranges. *p < 0.05 (Mann–Whitney *U* test)

In the acute phase of SAH, we found a significant increase in the total monocyte count compared to the chronic phase (p < 0.01). Among RAA patients compared to controls, the classical monocyte count was higher and, in comparison to RAC, both classical and intermediate monocyte counts were higher. The proportions of subsets also differed between the studied groups: among RAA subjects, the percentage of nonclassical monocytes was lower in comparison to controls, but the percentage of intermediate monocytes was higher compared to RAC patients. We did not observe significant differences between RAC and C groups (Fig. 4a). Dynamic changes were also observed in lymphocyte population.

Namely, their total count was lower in the acute phase of SAH compared to controls and the remote phase (p < 0.01). The same was true for CD3+ (T-lymphocytes) as well as CD4+ and CD8+ subsets. Among the investigated lymphocyte subpopulations, only the CD4+ percentage dropped significantly in acute SAH in relation to the remaining groups. Moreover, the CD4+/CD8+ ratio was significantly lower among RAA patients compared to controls (1.96 vs 2.66, respectively, p < 0.03) (Fig. 4b). The differences in the levels of lymphocytes were associated with the clinical outcome of the patients. The comparison of patients with Glasgow Outcome Scale scores of 4–5 vs 1–3 points indicated that lymphocytopenia is associated with a worse prognosis (p < 0.05). We did not observe any significant alterations in *i*NKT cell count (not shown).

To solve the question of whether observed changes in transcription levels are only related to the number of cellular sources, we analyzed alterations in cell-type specific transcripts. The transcripts specifically expressed in CD4+ and CD14+ cell populations were identified by using the mRNA abundance levels obtained from the BioGPS database (Human U133A/GNF1H GeneAtlas dataset). At the cellular level, the monocyte count increased 1.3-fold in RAA vs C subjects, whereas the CD14+-enriched transcripts showed a 1.8- to 4.6-fold increase in the RAA group. The CD4+ count was 1.5-fold lower in RAA vs C patients with a twofold drop in CD4+-enriched transcript levels. This could suggest that the observed changes in T cell-specific transcripts (at least CD4+) are mainly driven by cell number, whereas monocyte transcription is actively regulated in response to the pathological process.

### L/MN index

We tested a previously proposed L/MN index to evaluate prognosis in RAA patients [6]. The mean L/MN value was significantly higher in C compared with RAA patients (0.78 vs 0.24, respectively; p = 2.16E^−10^). The L/MN index was also associated with SAH severity: its value was significantly higher among RAA patients scored at 14–15 points on the Glasgow Coma Scale at admission compared with RAA patients scored at 3–13 points (0.26 vs 0.15, respectively; p < 0.05). When compared 2 patients who died with those who survived (n = 17), we observed nominally lower L/MN index among those with the worst outcome. However, the difference was not statistically significant. The mean L/MN index was insignificantly higher in RAC patients compared to C (0.88).

## Discussion

Rupture of an IA, particularly in the acute phase, causes noticeable changes in the composition of peripheral blood cells and their transcription profiles. The presented results suggest a depression of lymphocyte response and an enhancement of pro-inflammatory monocyte activity. These effects are no longer detectable several months after IA rupture. Similarly, van’t Hof et al. did not detect any significant alterations in gene expression profiles in blood cells among SAH survivors investigated years after IA rupture [16].

The analysis of transcription profiles revealed an increased abundance of transcripts related to activation of inflammatory response, Toll-like receptor signaling, and activation of innate immunity with a concomitant downregulation of transcripts related to positive leukocyte activation, T-cell differentiation and CD4+ and CD8+ lymphocytes. This is in line with our earlier findings [8]. Moreover, we confirmed that the previously proposed L/MN index (based on expression levels of lymphocyte-, monocyte- and neutrophil-related genes) is significantly lower in patients with SAH early after IA rupture compared to control subjects. A similar direction of changes was observed at the cellular level.

In acute SAH, the increase in overall monocyte count seemed to be driven mainly by the increased classical monocyte count. Similar changes were reported in myocardial infarction [17]. Analysis of the proportions of monocyte subsets showed that early after IA rupture, the percentage of classical monocytes did not change substantially, whereas the percentage of intermediate monocytes increased compared with controls, and nonclassical dropped compared to the chronic phase. These alterations resembled previously published observations [12]. The existing data regarding the influence of IA rupture on monocytes are conflicting. Both an increased count and activation of monocytes [18] as well as a lack of any significant changes were reported [6]. Our results suggest that in acute SAH, the number of monocytes increases with a preferential increase in classical monocyte count and—looking at the subset proportions—a decrease in nonclassical monocytes. Similar findings were reported in acute ischemic stroke [13]. In an experimental model, ischemic stroke was shown to enhance monocyte production by bone marrow stem cells [19, 20]. In humans, systemic inflammation caused release of classical monocytes from bone marrow and their subsequent maturation into intermediate and nonclassical cells [21]. Existing data suggest that classical monocytes first react to an inflammatory challenge and then they can mature into pro- or anti-inflammatory cells. The results of this study also suggest that IA ruptures elicit similar effects, i.e., release of classical monocytes followed by their transformation into other subclasses.

Simultaneously, we noticed significantly decreased levels of both CD4+ and CD8+ T-lymphocytes. A decrease in the CD3+ cell count within 24 h of IA rupture was previously reported [12]. However, CD4+ and CD8+ numbers did not differ significantly between the aneurysmal SAH subjects and controls. In the acute phase of other types of stroke, i.e., intracerebral hemorrhage and brain infarction, a decrease was found in the total lymphocyte count and in counts for the CD3+, CD4+, and CD8+ subsets [22, 23]. Decreased numbers of T-lymphocytes correlated with poor initial clinical status and worse outcomes, in different types of stroke [5, 11, 22–25]. Additionally, the analysis of TFBSs pointed at the shift towards pro-inflammatory reaction and deregulation of T-cells in the acute phase of SAH. Among the promoters of downregulated transcripts, we found significant overrepresentation for *STAT6* and *RUNX*. In monocytes, *STAT6* plays an important role in transcription of anti-inflammatory genes [26]. Runx proteins are critical for differentiation and function of CD4+ and CD8+ lymphocytes [27]. Lack of changes in the *i*NKT cell count after ischemic stroke was previously reported [28].

The systemic response to SAH is clearly a dynamic process. IA rupture predominantly evoked alterations in protein-coding transcripts. Interestingly, analysis of pre-mRNAs of differentially expressed genes in the acute phase revealed a lower exon/intron ratio among downregulated transcripts. This could suggest an attempt to enhance expression of genes whose total levels dropped due to a decreased number of cells from which they originate (probably T-cells). For a considerable subset of genes, we were able to identify alternatively spliced isoforms. Most of them were unique for the acute SAH. Similarly, specific mRNA isoforms in intracerebral hemorrhage and ischemic stroke of different etiologies were previously reported [29]. The functional significance of these transcriptional alterations remains unclear. *ELF2* (E74-like factor 2) plays an important role in lymphocyte development. Two *ELF2* isoforms derived from alternative promotors have been shown to exert opposite effects on cell proliferation. Namely, ELF2B had a negative influence on cell cycle progression and survival and induced apoptosis, and these negative effects were dependent on the presence of the N-terminus [30]. Here, we found that some of the *ELF2* transcripts had opposite directions of expression changes in acute SAH compared to the chronic phase. The isoform upregulated in RAA is built from the initial 7 exons, whereas the downregulated transcript, possesses the terminal 6–7 exons. Another identified gene with different isoform expression is *ACBD6* (acyl-coenzyme A binding domain-containing member 6). It is expressed predominantly in hematopoietic progenitor cells and is important for lipid homeostasis [31, 32]. Here, only isoforms with alternative splicing affecting the C-terminus, which contains ankyrin-repeat motifs, showed lower expression in RAA. These repeats mediate protein–protein interactions. For *HEATR1* (HEAT repeat-containing protein 1), we also found that distinct isoforms may have different expression levels in different periods after IA rupture. HEATR1 protein is involved in the regulation of ribosomal biogenesis [33]. Downregulation of HEATR1 causes G1-cell cycle arrest [34]. Here, one of the *HEATR1* transcripts was downregulated among RAA patients. Mutations within *ELF2* and *HEATR1* were identified in T-cell leukemia/lymphoma patients [35].

The presented results demonstrated that IA rupture causes multidirectional alterations in transcriptomes of peripheral blood cells. In ischemic stroke, a stroke-induced immunodeficiency syndrome is a recognized phenomenon, which involves reduced T-lymphocytes counts and enhanced apoptosis of immune cells in the spleen and thymus [36, 37]. Altogether, these results strongly suggest that the immune system responses to an acute vascular event are underlined by many common mechanisms. The significance of immunodepression is unclear. On the one hand, it is associated with more severe brain injury and an increased risk of infections. However, it could play a positive role in limiting the inflammatory response in the brain and reducing the immune reaction against brain-derived autoantigens [38]. Interestingly, analysis of alterations in cell numbers and transcripts levels suggest that changes in monocyte-specific transcripts are actively regulated and those related to lymphocytes depend on cell counts in acute SAH.

Our study has some potential limitations. One is the limited sample size and lack of validation of obtained results in another group of patients. However, to some extent, the present study is an extension of our previous work and the results are clearly parallel. We were able to confirm the specificity of the L/MN index. Next, for the transcriptomics, we used total peripheral leukocytes. Analyses performed in specific cell subclasses would provide a more comprehensive insight into the systemic response to IA rupture. Such an approach would also require a substantially larger blood sampling, and a problem regarding how the cell sorting procedure (to isolate cell subpopulations) could influence gene expression remains. In addition, we are aware that our study favored inclusion of RAA patients admitted in relatively good clinical condition. For the flow cytometry procedures only freshly obtained blood samples within working hours of the laboratory were useful. Before any study-related procedures were started we needed to obtained an informed consent—in almost all cases these consents were given by patients themselves. That means that patients admitted in the afternoon, in the evening, or at nights, holidays, etc. who required immediate interventions could to be included into the study.

## Conclusions

In summary, the results of this study demonstrated that IA rupture evokes a prominent systemic response clearly influencing the transcription profiles of peripheral blood cells as well as the composition of mononuclear cells. A specific pattern of gene expression alteration found here suggests a depression of lymphocyte response and enhancement of monocyte activity. These alterations are seen both in the cellular composition and in the gene expression profiles.

## Additional files

**Additional file 1: Table S1.** Validation of different biotypes of transcriptional variants regulated in response to IA rupture. Selected transcriptional variants (from the list included in Additional file 4: Table S3) were validated by using qPCR. The results are presented as the fold change with respect to the control group with a standard error of the mean (n = 10 to 14). Significant differences in the main effects from one-way ANOVA for the clinical status factor.

**Additional file 2: Table S2.** The specific IA-altered transcriptional variants of genes used for L/MN index calculation. The L/MN index was calculated as the ratio of the mean folds of standardized expression levels of lymphocyte-related genes (*BCL11B, CCR7, CD2, CD27, CD3D, CD3E, CD8A*, and *KLRB1*) to those of monocyte-and-neutrophil-related genes *(ANXA3, ARG1, CD14, GYG1, FCGR1A, FCGR2A, IRAK3, and MMP9*). L = lymphocyte-specific gene; M = monocyte-specific gene.

**Additional file 3: Figure S1.** Time-course of alterations in expression of *BASP1*-*AS1* and *SLC26A8* after the rupture of an intracranial aneurysm. Y-axis represents gene expression level for *BASP1*-*AS1* and *SLC26A8* presented as log2 FPKM values. On x-axis mean expression level measured in RAA samples, levels from RAC samples collected at specific time points and mean level measured in C samples are presented, respectively. The association between gene expression and time period of sample collection after the rupture were measured by Spearman’s rank correlation coefficient. The correlation coefficient was calculated as R = -0.76 (p < 0.0025) for *BASP1*-*AS1* (ENST00000399760) and R = -0.72 (p = 0.0048) for *SLC26A8* (ENST00000486155).

**Additional file 4: Table S3.** ANOVA results of expression profiling of transcripts regulated in response to IA rupture in peripheral blood. A table summarizing the results of the one-way ANOVA (FDR < 0.001) for the clinical status as the factor (RAA, RAC, C). The table contains the fold change of difference between the groups for each regulated transcript. Transcripts were annotated using the Ensembl gene database.

**Additional file 5: Table S4.** The results of the functional enrichment analyses performed with the Enrichr tool. WikiPathways, GO Biological processes, ChEA and GeneAtlas categories for the up- and downregulated genes in response to IA-rupture are included. The table consists of an enriched term, the number of input genes in the pathway (overlap), the p value (p < 0.05), the z score, the combined score (computed as logarithm from p value from the Fisher’s exact test multiplied by the z score of the deviation from the expected rank), and the names of overrepresented genes.

**Additional file 6: Table S5.** The results of pair-wise differential expression analysis of samples in each group using Cuffdiff. Three comparisons are presented: C vs RAA; C vs RAC, RAA vs RAC. At the transcript-level 148 differential expressed units were identified (q-values < 0.05). The analyses at the levels alternative exon splicing and alternative promoter usage did not provide significant results.

## Abbreviations

ACBD6: acyl-coenzyme A binding domain-containing member 6
C: control subjects
ELF2: E74-like factor 2
FDR: false discovery rate
HEATR1: HEAT repeat-containing protein 1
HPRT1: hypoxanthine–guanine phosphoribosyltransferase 1
IA: intracranial aneurysm
iNKT cell: invariant natural killer T cell
L/MN index: lymphocyte-to-monocyte-and-neutrophil index
NGS: next-generation sequencing
qPCR: quantitative polymerase chain reaction
RAA: acute ruptured aneurysms
RAC: chronic ruptured aneurysms
SAH: subarachnoid hemorrhage
TFBSs: transcription factor binding sites
WBC: white blood cell
