# Supplementary material for: Systemic response to rupture of intracranial aneurysms involves expression of specific gene isoforms
Source: J Transl Med. 2019 May 2;17:141. doi: 10.1186/s12967-019-1891-6 (PMC6498486; doi:10.1186/s12967-019-1891-6)
Supplement: Supplementary file 3 — Additional file 3: Figure S1. Time-course of alterations in expression of BASP1-AS1 and SLC26A8 after the rupture of an intracranial aneurysm. Y-axis represents gene expression level for BASP1-AS1 and SLC26A8 presented as log2 FPKM values. On x-axis mean expression level measured in RAA samples, levels from RAC samples collected at specific time points and mean level measured in C samples are presented, respectively. The association between gene expression and time period of sample collection after the rupture were measured by Spearman’s rank correlation coefficient. The correlation coefficient was calculated as R = -0.76 (p < 0.0025) for BASP1-AS1 (ENST00000399760) and R = -0.72 (p = 0.0048) for SLC26A8 (ENST00000486155). [file 12967_2019_1891_MOESM3_ESM.pdf]

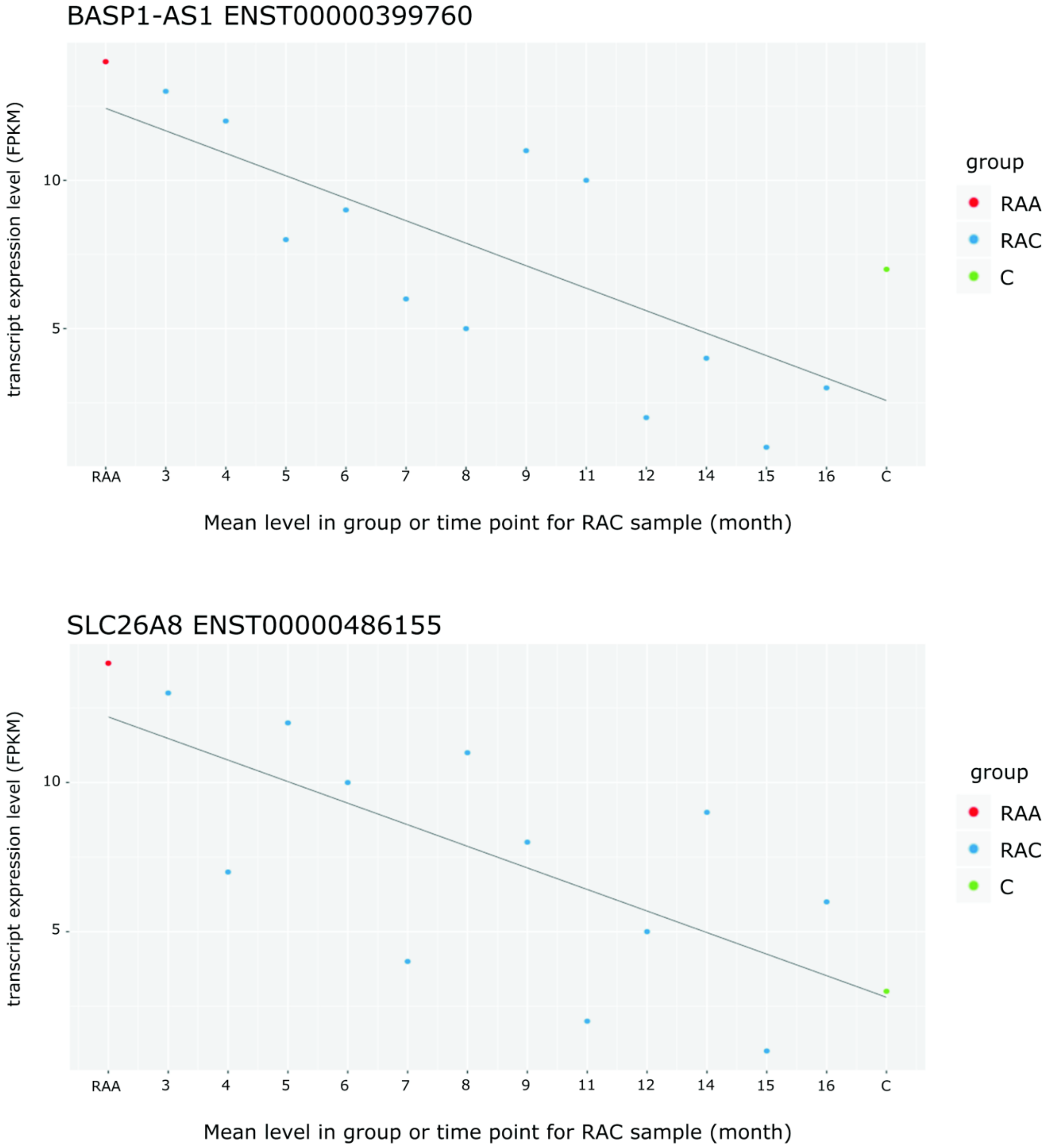

Time-course of alterations in expression of *BASP1-AS1* and *SLC26A8* after the rupture of an intracranial aneurysm.

Y-axis represents gene expression level for *BASP1-AS1* and *SLC26A8* presented as log2 FPKM values. On x-axis mean expression level measured in RAA samples, levels from RAC samples collected at specific time points and mean level measured in C samples are presented, respectively. The association between gene expression and time period of sample collection after the rupture were measured by Spearman's rank correlation coefficient. The correlation coefficient was calculated as R=-0.76 (p<0.0025) for *BASP1-AS1* (ENST00000399760) and R=-0.72 (p=0.0048) for *SLC26A8* (ENST00000486155).
